# Testing System for the Mechanical Properties of Small-Scale Specimens Based on 3D Microscopic Digital Image Correlation

**DOI:** 10.3390/s20123530

**Published:** 2020-06-22

**Authors:** Xu Liu, Rongsheng Lu

**Affiliations:** School of Instrument Science and Opto-electronic Engineering, Hefei University of Technology, Hefei 230009, China; liuxu0918@mail.hfut.edu.cn

**Keywords:** stereo light microscope, camera calibration, shape function, undermatching and overmatching, digital image correlation, micro tensile machine

## Abstract

The testing of the mechanical properties of materials on a small scale is difficult because of the small specimen size and the difficulty of measuring the full-field strain. To tackle this problem, a testing system for investigating the mechanical properties of small-scale specimens based on the three-dimensional (3D) microscopic digital image correlation (DIC) combined with a micro tensile machine is proposed. Firstly, the testing system is described in detail, including the design of the micro tensile machine and the 3D microscopic DIC method. Then, the effects of different shape functions on the matching accuracy obtained by the inverse compositional Gauss–Newton (IC-GN) algorithm are investigated and the numerical experiment results verify that the error due to under matched shape functions is far larger than that of overmatched shape functions. The reprojection error is shown to be smaller than before when employing the modified iteratively weighted radial alignment constraint method. Both displacement and uniaxial measurements were performed to demonstrate the 3D microscopic DIC method and the testing system built. The experimental results confirm that the testing system built can accurately measure the full-field strain and mechanical properties of small-scale specimens.

## 1. Introduction

With the rapid development of micro-electro-mechanical systems (MEMS), the mechanical properties of the materials used in MEMS are directly related to the design and reliability of MEMS products. When the sample size is reduced to the micro–nano level, the materials will exhibit specific properties, such as a large increase in intensity [1,2,3]. It is not reasonable to simply reduce the mechanical properties of the material under the conditions of macroscopic dimensions to those of small-scale specimens, so many traditional methods and instruments for testing the mechanical properties of materials will no longer be applicable [4]. At present, there are many testing methods for investigating the mechanical properties of small-scale specimens, including the beam bending method [5,6], the direct tensile method [7], the nano indentation method [8], the tympanic membrane method [9], and the test method for integrating the test piece with the test structure [10,11]. The beam bending method can be used for the testing of small test pieces, but the loading force is small. The nano indentation method is a contact test method, which causes a certain degree of damage to the test piece, and the calculation of mechanical property parameters is complicated. The residual stress in the tympanic membrane method must be tensile stress, and the interpretation of the experimental results is more complicated. The test piece and test structure integration test method integrates the test piece and the test structure. Although the resolution of force and displacement detection is higher, the test results include the performance of the test structure, so it cannot directly reflect the mechanical properties of the test piece and the processing of the test piece is extremely complicated. The direct tensile method directly records the Young’s modulus, yield strength, and breaking strength of the specimen by recording the stress–strain curve of the specimen’s tensile deformation. Of all the methods listed above, the results obtained with this method are the most accurate. The overall size of the specimen is at the millimeter level. Therefore, if we intend to perform a tensile test of a specimen, the following issues must be carefully considered: how to hold and load the test piece, how to obtain the true strain of the test piece, and so on.

The deformation measurement of small-scale specimens is key to the direct tensile method, and directly affects the accuracy of the obtained mechanical performance parameters. The digital image correlation method (DIC) has the advantages of being a non-contact approach and having a high accuracy and low environmental and equipment requirements, and has become the most active method in the field of optical measurement. It is widely used for measuring the shape, displacement, and full-field deformation of objects [12,13]. With the help of a stereo light microscope (SLM) vision system, digital image correlation methods can be applied to deformation measurement in small-scale areas [14,15,16,17].

Stereo microscopes have the characteristics of relatively complicated imaging optical paths and a small depth of field, so the calibration method employed for macroscopic stereo vision systems is no longer applicable. According to the imaging model, micro-calibration is mainly divided into two categories. Some calibration methods are based on the weak parallax imaging model. For example, Wang et al. [18] proposed a weak parallax-based micro imaging model based on the research of Kim [19] and Danuser [20]. The parameters, such as the magnification of the dual optical path and the angle between the main optical axis and the centerline, are obtained, but the model is more approximate. The others are based on the pinhole imaging model and combined with the traditional calibration method. Schreier et al. [21] proposed a complex light microscope stereo vision calibration method. It is implemented by firstly calculating the residual distortions of each point in the image plane to compensate for the distortion of the calibrated image, and then using Zhang’s calibration method [22] to obtain the system parameters. Ren et al. [23] approximated the imaging center area as an undistorted area in their investigation of 3D deformation measurement with the DIC method and a stereo microscope. The distortion model was presented by a B-spline surface. To calibrate the stereo microscope, an initial value was calculated by Tsai’s two-step method [24] and the final result was obtained by the Levenberg–Marquardt algorithm. The difficulty of this method is that the undistorted area is hardly determined.

The deformation measurement method that is based on digital image correlation needs to use a suitable shape function to characterize the true deformation of the target image sub-region. So far, most researchers have used first-order shape functions to approximate the deformation of the target image sub-region [25,26]. Compared with the second-order and even higher-order shape functions, the order shape function includes a small amount of calculation, and can basically accurately describe the deformation of the target image sub-region. However, for some more complex deformations, the first-order shape function describing uniform deformation is not good enough to approximate the non-uniform deformation of the target image sub-region. It is then necessary to use the second-order shape function and even higher-order shape functions [27,28]. Gao [27] analyzed the impact of different shape functions on the accuracy of crack-board deformation. The dimensions of the crack-board are not given in the paper. However, we can ascertain that the size of the crack-board should be in the order of 100 mm by observing the figures in the text. Inappropriate shape functions will cause undermatching or overmatching, which will cause large matching errors [29,30]. Wang [30] also conducted a simulated secondary deformation experiment and analyzed the reliability of the second-order shape function. Other than the use of different order shape functions, choosing the appropriate subset, step, and strain filter can also affect the accuracy of deformation measurement. Koohbor [31] optimized the main image correlation parameters, i.e., the subset, step, and filter size, through sensitivity analyses to facilitate accurate quantitative meso-scale strain deformation measurements. Rajan [32] provided guidelines for selecting DIC parameters in order to maximize the correlation and minimize the errors in displacement and strain.

In this paper, a testing system that is based on a 3D microscopic DIC method combined with a micro tensile machine is investigated. It can be used to measure the 3D shape, full-field deformation, and mechanical properties of small objects. In the following sections, Section 2 first interprets the micro tensile machine, which is used to clamp and stretch the small-scale specimen. Then, an improved calibration method and DIC method are described in detail. Section 3 discusses the effect of different shape functions on the matching accuracy, and some numerical experiments are presented. Section 4 presents the experimental validation of this test system based on 3D microscopic DIC combined with a micro tensile machine. Section 5 consists of the conclusions.

## 2. System Specification

**Figure 1:**
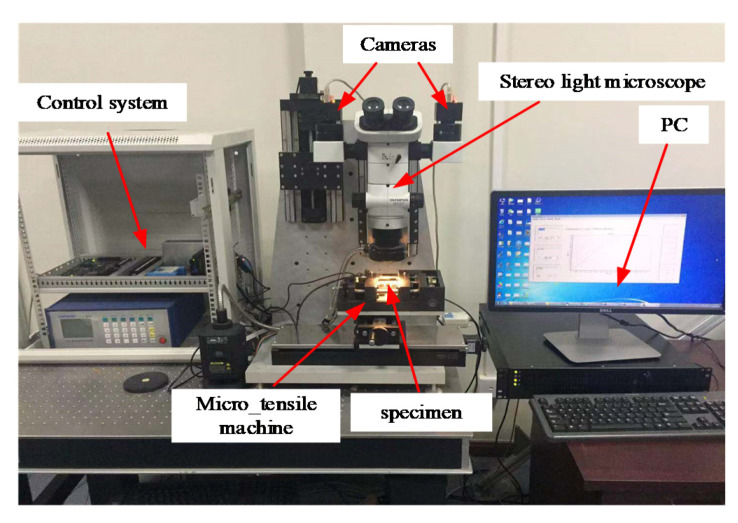
Testing system for investigating the mechanical properties of small-scale specimens based on 3D microscopic digital image correlation.

Figure 1 shows the hardware components of a testing system for investigating the mechanical properties of small-scale specimens based on 3D microscopic digital image correlation, mainly including the following: (1) SZX07 COM SLM (including an external ring light source, Olympus, Tokyo, Japan); (2) two industrial cameras (1280 pixels × 1024 pixels, DAHENG IMAGING, Beijing, China); (3) a micro tensile machine (self-made); (4) a control system (PMAC Clipper is the core of the control system, Delta Tau, Lassen St. Chatsworth, CA, United States); (5) and a PC (i7-6700HQ, 2.60 GHz, 8 GB, HP, Palo Alto, California, United States) for testing software installation. Firstly, we describe the micro tensile machine for holding and loading the specimen.

### 2.1. Micro Tensile Machine

**Figure 2:**
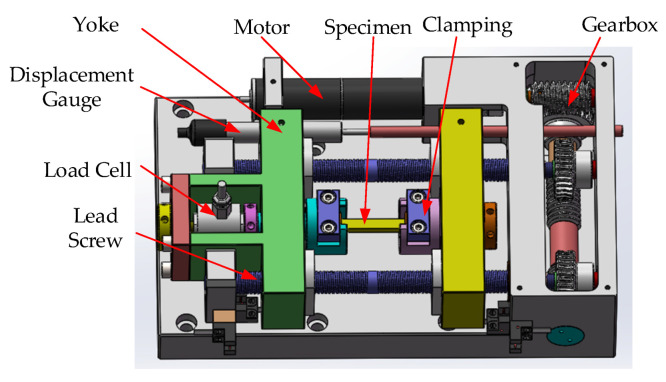
Elements of the micro tensile machine.

As shown in Figure 2, the micro tensile machine has two parallel yokes, with guide holes between which the specimen is drawn apart. Two lead screws have threads of the opposite pitch at their ends. These lead screws turn in strict synchronism. With the opposite direction of the pitch at the ends, this movement makes the yokes move towards or away from each other. One of the yokes holds the load cell (0–200 N) in a rigid, perpendicular position. This load cell can have different load ranges, and its choice demands selecting the electronic measuring boards. Additionally, each cell must be combined with a matching board inside the controller. The displacement gauge parallel to the lead screws is mounted at the side of the yokes. The plunger of the linear variable differential transformer (LVDT, −12.5 ± 12.5 mm) captures the addition of the movement of both yokes, thus reading the true change in the length of the specimen during the experiment. The lead screws are turned by the motor, down geared at a great ratio from the shaft to the lead screw. Therefore, the micro tensile machine can stretch very slowly and in small amounts. It is very helpful for us to study the deformation process of the specimen, and the specific performance parameters are shown in Table 1. Since the specimen needs to be clamped to the tensile machine, the specimen size has certain restrictions. Considering that both ends of the sample need to be tightened and fixed by the clamping jaw block, the minimum length of the specimen is generally more than 5 mm. For a particularly small specimen, you can use a special fixture to complete the tensile test. For brittle samples, high-strength glue can be used to bond materials with a good toughness at both ends of the specimen, and then install them on a tensile machine for tensile experiments.

Most of the existing tensile machines are fixed at one end, and the sample is stretched toward the other end [33]. The advantages of the tensile machine designed in this paper are mainly as follows: (1) with the opposite direction of the pitch at the ends, the transmission efficiency is improved and the transmission distance is reduced by half; (2) the lead screws turn in strict synchronism, which can ensure that no distortion will occur during the stretching process; and (3) the two-stage gear and worm meshing transmission are used to make the tensile machine compact in structure and low in vibration, and ensure that it is easy to prevent the reverse and achieve self-locking, which can effectively avoid accidental fracturing of the specimen.

### 2.2. Imaging System

**Figure 3:**
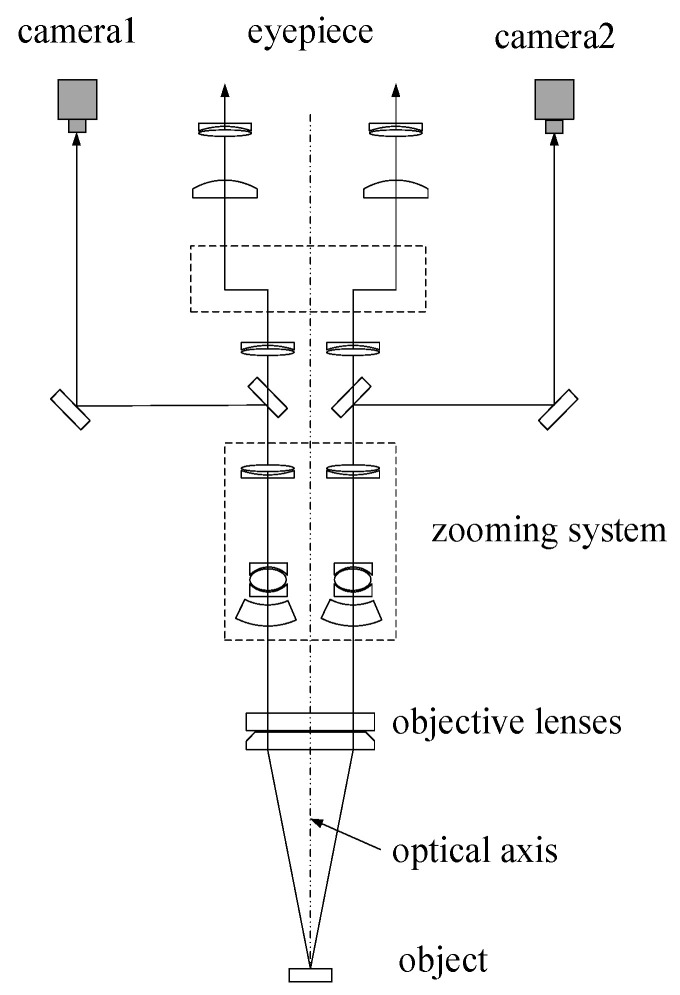
Schematic diagram of the light path of a common main objective-stereo light microscope (COM-SLM; ref. [34], Figure 1).

The imaging system consists of two cameras and a stereo light microscope. Greenough SLM (G-SLM) and the common main objective (COM) SLM are widely used. The COM-SLM was selected as part of the image system, because it has more advantages than G-SLM. For example, the image plane of COM-SLM is approximately parallel to its focal plane and COM-SLM requires less installation space. As shown in Figure 3, the light beam reflected from the inspected object is collected by the objective lenses, magnified by the zoom system, and then divided into two optical paths. Each partial beam is further split into two beams. One passes through the eyepiece lens system and the other reaches the digital cameras, where the stereo images are viewed by human eyes or captured by the two cameras. The SLM optical configuration obviously shows that the stereo imaging effect is the same as that of a binocular vision system.

#### 2.2.1. Model of the Imaging Optical Path

The traditional pinhole camera model can be used to describe the positional relationship between the object and its image. $\hat{m}={\left[u,v,1\right]}^{T}$ and $\hat{X}={\left[X_{w},Y_{w},Z_{w},1\right]}^{T}$ are homogeneous coordinates of the point P in the camera coordinate system o-xyz and image coordinate system O-XY, respectively. Furthermore, the relationship between them can be given by Equation (1) [22]:

(1) $$s\tilde{m}=A\left[\begin{array}{cc} R & T \end{array}\right]\tilde{X},$$

where s is an arbitrary scale factor. *A* and $\left[\begin{array}{cc} R & T \end{array}\right]$ are the intrinsic matrix and extrinsic matrix, which can be described as

(2) $$A=\left[\begin{array}{ccc} s_{x}\cdot f/d_{x} & \vartheta & u_{0}\\ 0 & f/d_{y} & v_{0}\\ 0 & 0 & 1 \end{array}\right],\;R=\left[\begin{array}{ccc} r_{1} & r_{2} & r_{3}\\ r_{4} & r_{5} & r_{6}\\ r_{7} & r_{8} & r_{9} \end{array}\right],\;T=\left[\begin{array}{c} T_{1}\\ T_{2}\\ T_{3} \end{array}\right],$$

where f, $d_{x}$, $d_{y}$, ϑ, $\left(u_{0},v_{0}\right)$, and $s_{x}$ are intrinsic parameters which represent the focus length; center to center distances between adjacent sensor elements in X and Y directions, respectively; the skewness coefficient of two axes; the image scale factor; and the computer image coordinates of the principal point, respectively. $r_{i}$ (*i* = 1, 2,…, 9) and $T_{j}$ (*j* = 1,2,3) are rotation and translation parameters, respectively.

The optical path of COM-SLM is more complicated than that of thin lenses. There are many factors that cause distortion, including the imperfect production of lenses and deviation in the installation.

The distortion of the CMO-SLM is non-linear. It is impossible to describe this complex distortion well with the first-order or higher order radial distortion model. In this paper, a non-parametric distortion model of the imaging plane is constructed to correct the distorted images. Moreover, the specific process is referred to in ref. [34].

#### 2.2.2. Calibration Process

We have confirmed that the iteratively weighted radial alignment constraint method is feasible for the calibration of SLM and the distorted images can be corrected by the distortion model, so we made a few changes to the calibration process. The modified discrete implementation steps are listed below:

1. Calibrate the imaging system by the weighted radial alignment constraint method and construct a non-parametric distortion model. The specific calibration steps can be found in detail in the ref. [34];
2. Revise (*u*, *v*) according to the constructed distortion model and repeat step 1;
3. If the number of iterations reaches the set value, terminate the iteration; otherwise repeat step 1 and 2. The parameter corresponding to the minimum reprojection error is output as the result.

### 2.3. Digital Image Correlation

#### 2.3.1. 2D-DIC Method with ZNCC+IC-GN

**Figure 4:**
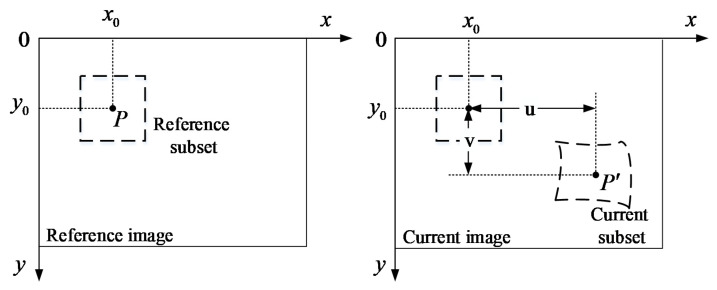
Principle graph of the two-dimensional digital image correlation (2D-DIC) method.

The digital image correlation method was used to compare digital images, so as to determine the displacement, deformation, or other factors. Figure 4 schematically shows a typical two-dimensional DIC (2D-DIC) method. A rectangular area around point P (x_0_, y_0_) in the reference image was selected as the reference subset, and the position of it in the current image was to be found. A typical correlation function zero-normalized cross-correlation (ZNCC), which is insensitive to linear changes of a gray image, is usually used to evaluate the correlation between the reference and current subset [35]. As an equivalent, but more efficient, strategy [36,37,38], the inverse compositional Gauss–Newton (IC-GN) algorithm combined with the ZNCC correlation function has been used for sub-pixel matching. The details of the IC-GN algorithm can be referred to in ref. [38].

Let W(x,y;p) denote the shape function that reflects the mapping rule between the pixel coordinates of points on the reference image and those of the corresponding points in the current image. The gray levels of the reference and current subsets are respectively denoted by T and I, and their corresponding mean intensity values can be denoted by $\bar{T}$ and $\bar{I}$.

The ZNCC criterion can be described as

(3) $$C_{ZNCC}=\sum_{y=-M}^{M}\sum_{x=-M}^{M}\frac{\left[T(W(x,y;\Delta p))-\bar{T}\right]\left[I(W(x,y;p))-\bar{I}\right]}{T_{s}I_{s}},$$

(4) $$T_{s}=\sqrt{\sum_{y=-M}^{M}\sum_{x=-M}^{M}{\left[T(x,y)-\bar{T}\right]}^{2}},$$

(5) $$I_{s}=\sqrt{\sum_{y=-M}^{M}\sum_{x=-M}^{M}{\left[I(x,y)-\bar{I}\right]}^{2}}.$$

In order to obtain the value of p, we applied the first-order Taylor expansion to the changed reference subset and obtained the following expression:

(6) $$C_{ZNCC}=\sum_{y=-M}^{M}\sum_{x=-M}^{M}\left[T(W(x,y;0))+\nabla T\frac{\partial W}{\partial p}\Delta p-\bar{T}\right]\left[I(W(x,y;p))-\bar{I}\right]/\left(T_{s}I_{s}\right).$$

The solution to the above least-squares problem is

(7) $$\Delta p=-H^{-1}\sum_{y=-M}^{M}\sum_{x=-M}^{M}{\left[\nabla T\frac{\partial W}{\partial p}\right]}^{T}\left[T(W(x,y;0))-\bar{T}-\frac{T_{s}}{I_{s}}I(W(x,y;p))+\frac{T_{s}}{I_{s}}\bar{I}\right],$$

where **H** is the Hessian matrix:

(8) $$H=\sum_{y=-M}^{M}\sum_{x=-M}^{M}{\left[\nabla T\frac{\partial W}{\partial p}\right]}^{T}\left[\nabla T\frac{\partial W}{\partial p}\right].$$

Then, W(x,y;p) can be updated by

(9) $$W(x,y;p)=W(x,y;p)W^{-1}(x,y;\Delta p).$$

#### 2.3.2. 3D-DIC Method

The three-dimensional DIC (3D-DIC) method is based on the 2D-DIC method and combines the stereo vision measurement technology. Two image sequences of an object were captured from different view angles by camera 1 and camera 2. The 2D-DIC method and stereo matching method were used to find the pixel coordinates of the image points of the object in the left and right image sequences. According to the positional relationship between the two cameras, the 3D coordinates of the surface points of the object could be calculated from their pixel coordinates.

**Figure 5:**
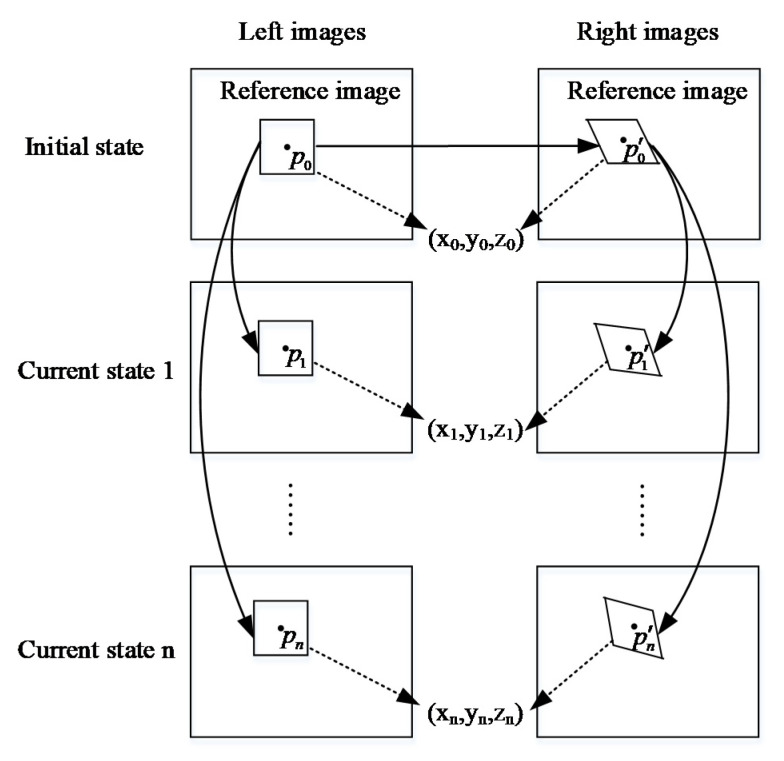
A schematic overview of three-dimensional digital image correlation (3D-DIC).

There are about three kinds of matching strategies that can be employed to find the pixel coordinates of image points in the left and right image sequences. Figure 5 shows the one with the highest accuracy. Images captured in the initial state are respectively set as the left and right reference images. Without a loss of generality, we can assume that the image point $p_{0}$ in the left reference image is corresponding to point P on the object surface. The corresponding image point ${p^{\prime }}_{0}$ in the right reference image can be found. After that, the pixel coordinates of all the image points of point p in the left image sequence were calculated by comparing them to the image point $p_{0}$ in the left reference image. Moreover, the pixel coordinates of all the image points of point P in the right image sequence were calculated by comparing them to the image point ${p^{\prime }}_{0}$ in the right reference image.

### 2.4. Testing Progress

Before starting the tensile test, some preparations need to be undertaken. According to the size of the specimen, appropriate magnification of the COM-SLM was chosen to ensure that the deformation area of the specimen was in the field of view during the entire test. Furthermore, the imaging system was calibrated as described in Section 2.2.2. After the calibration was completed, the small-scale specimen was installed and drawn apart, while the load value and images were collected by the test system. When the specimen was fractured, the micro tensile machine stopped working, as do the cameras. In the image of the initial state, an area (or several areas) was selected as the reference area. Several grid points evenly distributed in the selected area were chosen as sample points. The 3D coordinates of the selected grid points in all states were calculated by the 3D microscopic DIC method, which is interpreted in Section 2.3.2. Then, the full-field strain and mechanical properties of the specimen could be calculated based on the above data.

## 3. Effects of Shape Functions on the Matching Accuracy

In Section 2.3.1, the principle of the ZNCC+IC-GN algorithm was described in detail. Choosing a suitable type of shape function to represent the true deformation or displacement value is directly related to the matching accuracy. In this section, we first listed three kinds of shape functions. Then, we used a computer to generate a speckle image, and simulated the rigid body displacement, rotation, uniform deformation, and non-uniform deformation of the speckle pattern, in order to verify the effect of the shape function on the matching accuracy.

### 3.1. Shape Function

The zero-order, first-order, and second-order shape functions $W_{0}\left(x,y;p_{0}\right)$, $W_{1}\left(x,y;p_{1}\right)$, and $W_{2}\left(x,y;p_{2}\right)$ can be respectively described in the matrix form as follows:

(10) $$W_{0}\left(x,y;p_{0}\right)=\left[\begin{array}{c} x^{\prime }\\ y^{\prime } \end{array}\right]=\left[\begin{array}{ccc} 1 & 0 & u\\ 0 & 1 & v \end{array}\right]\left[\begin{array}{c} x\\ y\\ 1 \end{array}\right],p_{0}={\left[u,v\right]}^{T},$$

(11) $$W_{1}\left(x,y;p_{1}\right)=\left[\begin{array}{c} x^{\prime }\\ y^{\prime } \end{array}\right]=\left[\begin{array}{ccc} 1+u_{x} & u_{y} & u\\ v_{x} & 1+v_{y} & v \end{array}\right]\left[\begin{array}{c} x\\ y\\ 1 \end{array}\right],p_{1}={\left(u_{x},v_{x},u_{y},v_{y},u,v\right)}^{T},$$

(12) $$W_{2}\left(x,y;p_{2}\right)=\left[\begin{array}{c} x^{\prime }\\ y^{\prime } \end{array}\right]=\left[\begin{array}{cccccc} 1+u_{x} & u_{y} & \frac{1}{2}u_{xx} & u_{xy} & \frac{1}{2}u_{yy} & u\\ v_{x} & 1+v_{y} & \frac{1}{2}v_{xx} & v_{xy} & \frac{1}{2}v_{yy} & v \end{array}\right]\left[\begin{array}{c} x\\ y\\ x^{2}\\ xy\\ y^{2}\\ 1 \end{array}\right],\;p_{2}={\left(u,u_{x},u_{y},u_{xx},u_{xy},u_{yy},v,v_{x},v_{y},v_{xx},v_{xy},v_{yy}\right)}^{T}.$$

### 3.2. Numerical Experiments

#### 3.2.1. Rigid Body Translation and Rotation Experiments

**Figure 6:**
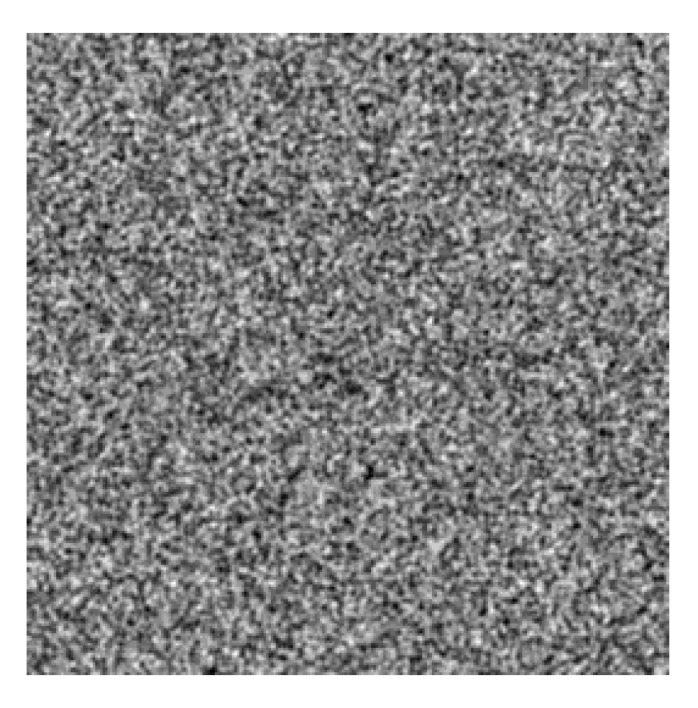
Speckle image generated by a computer.

Although the matching accuracy is related to the subset, only the effect of shape functions is discussed in Section 3.2. Figure 6 shows a speckle pattern with a size of 801 × 801, which was generated by Zhou’s method [39]. Then, twenty translated speckle images were investigated by the Fourier phase shift method [40], and the shift between successive images was 0.05 pixels. The ZNCC+IC-GN algorithms with zero-order, first-order, and second-order shape functions were respectively used to calculate the rigid body translation. The area of interest of about 450 × 450 was divided into grids with a step of 15 pixels. The subset size was N × N (N = 11, 21, 31, 41, 51, and 61), and the bicubic spline interpolation method was used to calculate the gray value at the sub-pixel position. The mean bias error epsilon and standard deviation sigma were used to evaluate the matching accuracy of the three shape functions. The bias error epsilon and standard deviation sigma can be described as

(13) $$\varepsilon =\left(1/N\right)\sum_{i=1}^{N}\left|x_{calc}-x_{set}\right|,$$

(14) $$\sigma =\sqrt{\left(1/\left(N-1\right)\right)\sum_{i=1}^{N}{\left(\left|x_{calc}-x_{set}\right|-\varepsilon \right)}^{2}}.$$

**Figure 7:**
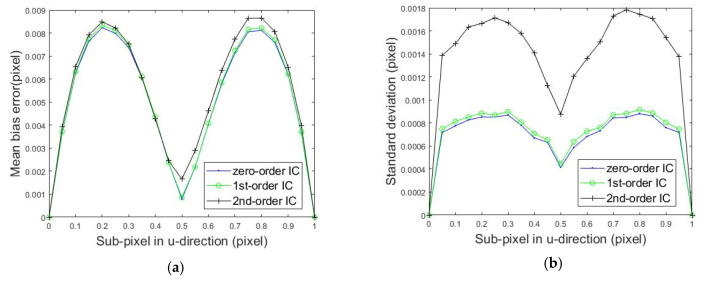
Calculated errors of the rigid body translation compared to the set sub-pixel values. (**a**) Mean bias error and (**b**) standard deviation.

Figure 7 shows the mean bias errors and standard deviation of rigid body translations measured by the IC-GN algorithm with three kinds of shape functions when the subset size was 31 × 31. In Figure 7a, it can be easily seen that the mean bias errors generated by employing the three shape functions were very close, and the error produced by employing the zero-order shape function was slightly smaller. As shown in Figure 7b, the standard deviation obtained by employing the second-order shape function was twice as large as that when using zero-order and first-order shape functions. Moreover, the computational speed of the IC-GN algorithm with the second-order shape function was less than half of the result of the IC-GN algorithm with the first-order shape function.

**Figure 8:**
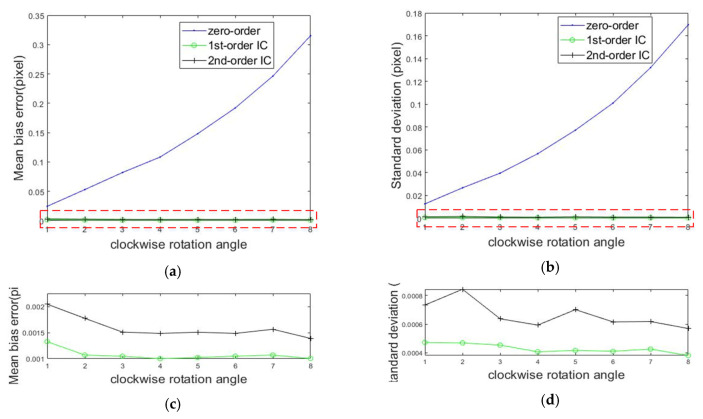
Calculated errors of the rigid body rotation compared to the set rotation angles. (**a**) Mean bias error; (**b**) standard deviation; and (**c**,**d**) enlarged views of the red dotted areas in (**a**) and (**b**), respectively.

The speckle image was rotated clockwise, and the rotation angle was increased by 1 degree each time. Then, ten rotated speckle images were obtained. The calculation steps were the same as those employed for the translated speckle images. Figure 8 shows the mean bias errors and standard deviations when the subset size was 31 × 31 and Figure 8c,d are enlarged views of the red dotted areas in Figure 8a,b, respectively. As shown in Figure 8a,c, the mean bias errors produced by employing first-order and second-order shape functions were about 0.001 and 0.0015 pixels. Additionally, the error induced by employing the zero-order shape function was much greater than the previous two, and the value became larger as the rotation angle increased. It is well-known that the first-order shape function can describe the rigid body rotation exactly. Therefore, an appropriate shape function will make the measurement more accurate.

#### 3.2.2. Uniform and Non-Uniform Deformation Experiments

**Figure 9:**
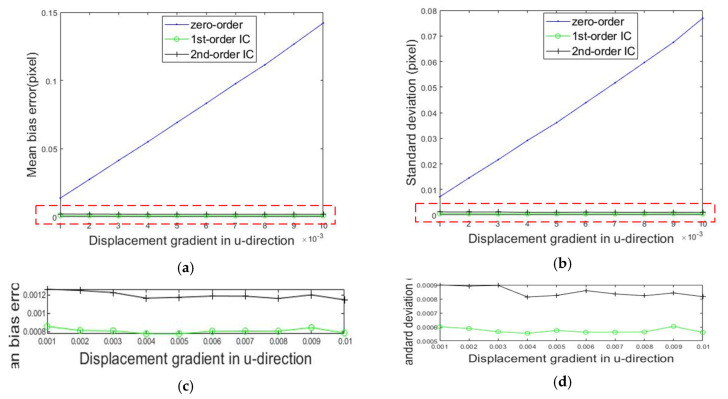
Calculated errors of the uniform deformations compared to the set values. (**a**) Mean bias error; (**b**) standard deviation; and (**c**,**d**) enlarged views of the red dotted areas in (**a**) and (**b**), respectively.

Ten uniformly deformed speckle images were generated by imposing a uniform strain of 0.01–0.1 on Figure 6 in the horizontal direction by the inverse mapping method. As in the rigid body translation test, ZNCC+IC-GN algorithms with three kinds of shape functions were used to calculate the displacements with uniform deformation. Figure 9 shows the mean bias errors and standard deviations when using a subset size of 31 pixels × 31 pixels and Figure 9c,d are enlarged views of the red dotted areas in Figure 9a,b, respectively. As shown in Figure 9, the experimental results were very close to those of the rigid body rotation experiments.

Next, an irregular load was added to the speckle image, and the deformation of each point conforms to the following formula (27):

(15) Δx=λTsin(2πx/T),

where λT and T denote the amplitude and period of the deformation, respectively. T is set to half of the width of the speckle image and λ is set to a range of 0.001–0.01, with an increment of 0.001. In this way, the size of the deformed image is the same as that of the original speckle image and each point on the original speckle image is deformed by no more than one pixel.

**Figure 10:**
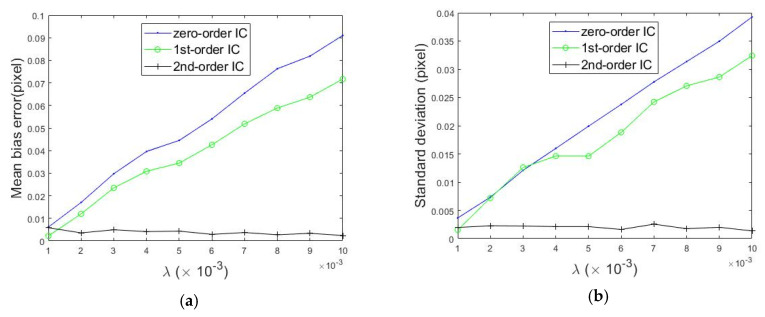
Calculated errors of the non-uniform deformations compared to the set values. (**a**) Mean bias error; (**b**) standard deviation.

Figure 10 shows the mean bias errors and standard deviation of non-uniform deformations compared to the set values measured by the IC-GN algorithm with three kinds of shape functions when the subset size was 31 × 31. In Figure 10a, it can easily be seen that with the increase of amplitude, the mean bias errors produced by employing zero-order and first-order shape functions increase to a large value approximately linearly, while the error produced by employing the second-order shape function is small and stable in a small range. As we expected, the second-order shape function can represent the non-uniform deformation better.

### 3.3. Discussion

According to the ways in which the numerical simulation speckle images were generated in Section 3.2, the following information can be easily obtained: (1) relative to rigid body displacement, both first-order and second-order shape functions are overmatched; (2) relative to rigid body rotation and uniform deformation, the zero-order shape function is undermatched, and the second-order shape function is overmatched; and (3) relative to non-uniform deformation, both zero-order and first-order shape functions are undermatched. It can be seen from the numerical experiments that (1) the mean bias error induced by an undermatched shape function is larger than that of an overmatched shape function; (2) the use of undermatched shape functions makes the measurement results more dispersive; and (3) an overmatched shape function will not cause greater errors, but the calculation speed will be severely reduced.

**Figure 11:**
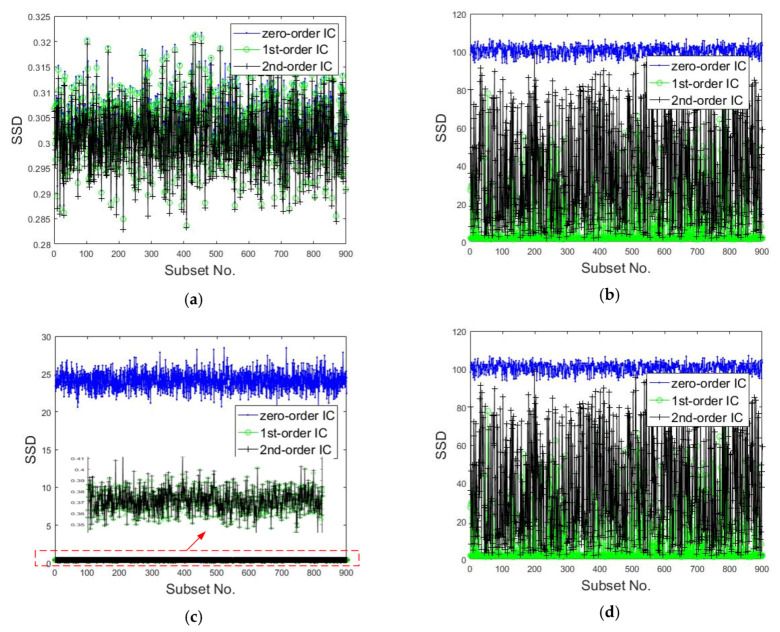
Sums of squared differences (SSDs) between the reference and current subsets calculated by DIC with three kinds of shape functions: (**a**) speckle image of rigid translation, (**b**) speckle image of rigid rotation, (**c**) speckle image of uniform deformation, and (**d**) speckle image of non-uniform deformation.

For the simplicity of calculation, the sums of squared differences (SSDs) between the reference subset and the best matching subset in the current images with three kinds of shape functions were also calculated. The SSDs of 900 subsets of different images are shown in Figure 11. The smaller the value is, the higher the accuracy of the matching will be. For instance, in Figure 8, Figure 9, and Figure 11b,c, the correlation coefficient SSDs calculated by IC-GN with first-order and second-order shape functions are close and much smaller than that ascertained with the zero-order shape function, and the corresponding result is that the accuracy of the measurements obtained by employing first-order and second-order shape functions is higher. According to the above analysis, the value of the correlation coefficient is reasonable as a criterion for selecting the shape function type.

According to the characteristics of the micro tensile machine introduced in Section 2.1, the small-scale specimen installed between two parallel yokes is in situ stretched in opposite directions. The deformation of the whole field is not uniform during the tensile test of the specimen. The amount of deformation near the fracture location is large, and that far from that location is small. If a single shape function is applied to meet the requirements of the IC-GN algorithm in the whole field, undermatched or overmatched problems will inevitably arise.

## 4. Experiments

### 4.1. System Calibration

**Figure 12:**
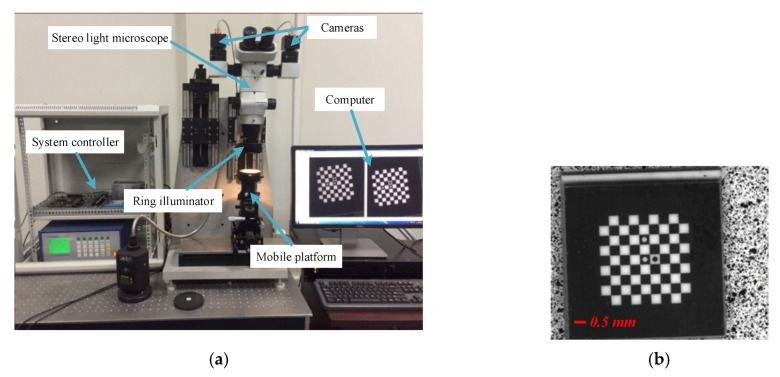
The calibration of a COM-SLM. (**a**) Calibration device and (**b**) target (ref. [34], Figure 5 and Figure 6).

As shown in Figure 12, Figure 12a,b is the calibration device and the target, respectively. The target is fixed on the mobile platform. The magnification of COM-SLM was adjusted to the appropriate position where the target can be clearly imaged. The imaging system was recalibrated by the modified iteratively weighted radial alignment constraint method described in Section 2.2. The calibration result can be seen in Table 2, and Table 3 shows the reprojection errors calculated by five different calibration methods. It can be seen that the modified calibration method can attain a higher accuracy and complete calibration of the system.

### 4.2. In-Plane Displacement Measurement

**Figure 13:**
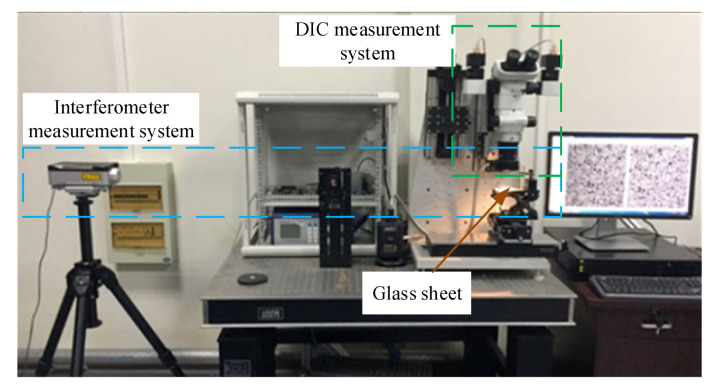
In-plane displacement measurement.

In-plane displacement measurement was performed to verify the 3D microscopic digital image correlation. Figure 13 shows the measurement setup. A glass sheet sprayed with a black and white speckle pattern was fixed on a linear platform and moved with a step of about 0.1 mm. One hundred marker points were generated when a region of 600 pixels × 600 pixels was divided into grids with a step of 60 pixels. For the spray painted speckles, 99% correlation was obtained at 17 pixels and full (100%) correlation at 29 pixels [32]. Therefore, we chose a subset of 31 pixels × 31 pixels. The 3D coordinates of these points were calculated by 3D microscopic DIC with first-order and second-order shape functions before and after movement. The average displacement of these points was recognized as the current displacement value. At the same time, the displacement of the movement was measured by a laser interferometer as the standard values for accuracy evaluation of 3D microscopic DIC with different kinds of shape functions.

**Figure 14:**
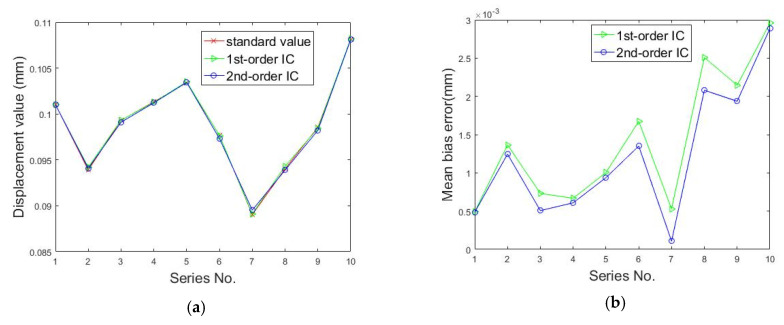
Displacements calculated by DIC with two kinds of shape functions with a fixed subset size of 31 pixels × 31 pixels: (**a**) displacement value and (**b**) mean bias errors.

As shown in Figure 14a, the displacements calculated by 3D microscopic DIC with first-order and second-order shape functions were very close to the standard values. The result could confirm that the 3D microscopic DIC and calibration method could achieve a small-scale measurement. The transformation between the two images captured by the left and right cameras can be generally considered as a plane-to-plane homography [41]. In Figure 14b, it can be easily seen that the mean bias errors of displacements calculated by employing the second-order shape function (about 0.0012 mm) were smaller than those calculated by employing the first-order shape function (about 0.0015 mm). The result could also confirm that the measurement accuracy of IC-GN with a second-order shape function was higher than that of IC-GN with a first-order shape function when we deal with the plane-to-plane homography [27].

### 4.3. Uniaxial Tensile Experiment

**Figure 15:**
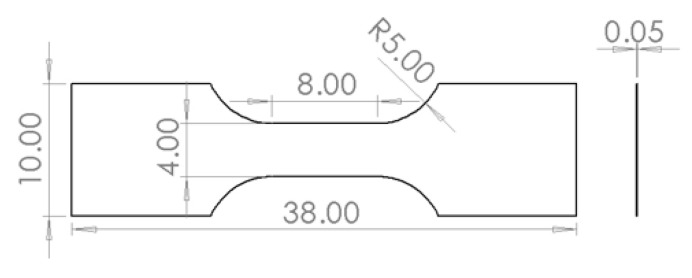
Specimen size.

The stress and strain of a T2 pure copper thin specimen was measured under uniaxial tension by using the system designed for testing the mechanical properties of small-scale specimens. As shown in Figure 15, the deformation area size of the specimen was 8 mm × 4 mm × 0.05 mm. By adjusting the lighting angle, the surface texture of the specimen already contained enough matching features, even if the surface was not sprayed with a black and white speckle pattern. This can eliminate the influence of the spray coating on the surface of the specimen on the measurement accuracy. Since the surface of the sample was not coated with speckles, it may be impossible to calculate the appropriate subset size according to the method presented in this article [32]. However, the change of the subset size directly affected the test accuracy of DIC strain measurements. Therefore, the ideal subset size needed to be evaluated according to the mean bias errors of the specimen rigid-body translation between the values calculated by the DIC method and the value measured by a laser interferometer.

**Figure 16:**
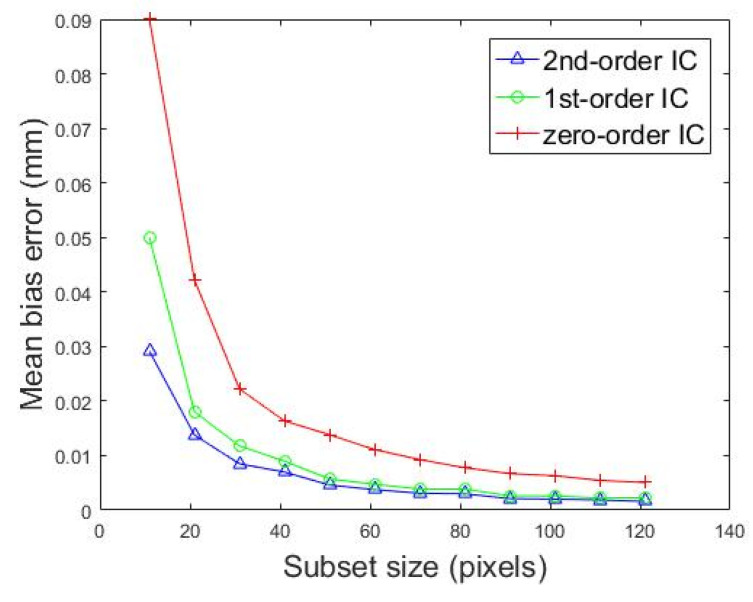
Calculated mean bias errors with different subset sizes using the DIC method by employing three kinds of shape functions.

The experimental setup is shown in Figure 13, and the only difference was that the glass sheet was replaced with the specimen, which was clamped in the micro tensile machine. Furthermore, the micro tensile machine was fixed on a linear stage. The specimen was moved with a step of about 0.1 mm by driving the above linear stage. One hundred and ten marker points on the specimen surface were selected with a step of 30 pixels. Twelve subset sizes were selected to calculate the displacement of the specimen using the DIC method by employing zero-order, first-order, and second-order shape functions. As shown in Figure 16, as the subset size increased, the mean bias error would gradually decrease. After the subset size reached 91 pixels, the mean bias error tended to be stable and did not decrease significantly as the subset size increased. No matter which shape function we chose, the above phenomenon was true. In addition, the curve in Figure 16 could also prove that selecting the second-order shape function was more conducive to improving the matching accuracy in stereo matching. We carefully observed the texture of the specimen surface. We assumed that the area of the sample surface with significantly smaller gray scales was speckled. At this resolution, the average speckle size *h_sp_* was below 30 pixels. With the rule of thumb for achieving optimal correlation, presented as $h_{sub}>3h_{sp}$ (where *h_sub_* represents the subset size) [42], the recommended subset size is 90 pixels. It can be concluded from the above analysis that the ideal measurement accuracy could be obtained when the subset size was 91 pixels. If the size is increased, the accuracy will not be significantly improved, but it will affect the calculation efficiency. After the subset size was determined, the small-scale specimen was installed between two parallel yokes with an in-situ stretching speed of 4 μm/s in opposite directions. The load data and images in different states were saved.

**Figure 17:**
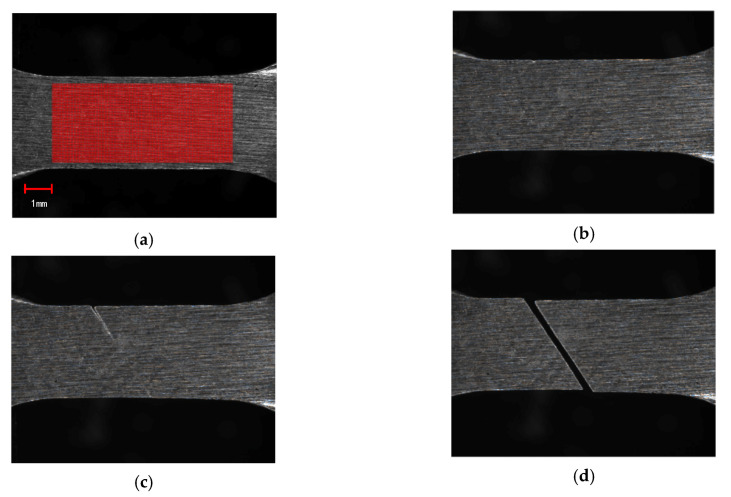
Images of the in-situ observation of a specimen. (**a**) Initial state, (**b**) elastic deformation stage, (**c**) necking stage, and (**d**) fracture state.

As shown in Figure 17, four images of the specimen captured by Camera 1 in different states are selected to illustrate the whole stretching process. Figure 17a shows the specimen in the initial state, and there were 11,424 points with the grid step of 5 pixels in the red marked area. Figure 17b shows that the specimen displays large deformation in the elastic state. Figure 17c,d are the images of the specimen before and after the fracture, respectively. The 3D coordinates of these points in the marked area in Figure 17a were calculated by the 3D microscopic DIC method with the subset size of 91 pixels × 91 pixels. Taking the displacement calculation of a single marker $p_{0}$ as an example, the specific calculation process is as follows: we first found the corresponding image point ${p^{\prime }}_{0}$ in the right reference image according to the IC-GN algorithm by employing the second-order shape function. The subset size of 91 pixels × 91 pixels around the image point $p_{0}$ was recognized as the reference subset in the left image sequences. Correspondingly, the subset size of 91 pixels × 91 pixels around the image point ${p^{\prime }}_{0}$ was recognized as the reference subset in the right image sequences. Then, we searched for the positions that matched the reference subset by the IC-GN algorithm by employing the first-order and second-order shape functions in the left and right image sequences, respectively. The ones with the larger absolute value of CZNCC were recognized as the matching points $p_{i}(i=1,2,\cdots ,n)$ and ${p^{\prime }}_{i}(i=1,2,\cdots ,n)$. The three-dimensional coordinates of the marked point were obtained according to the positional relationship between the two cameras. Compared with the in-plane displacement, the off-place displacement was extremely small during the stretching of the specimen. Therefore, the horizontal coordinate interpolations of the marked points were taken as the horizontal displacements. The strains were calculated using a two-dimensional Savitzky–Golay digital differentiator [43].

**Figure 18:**
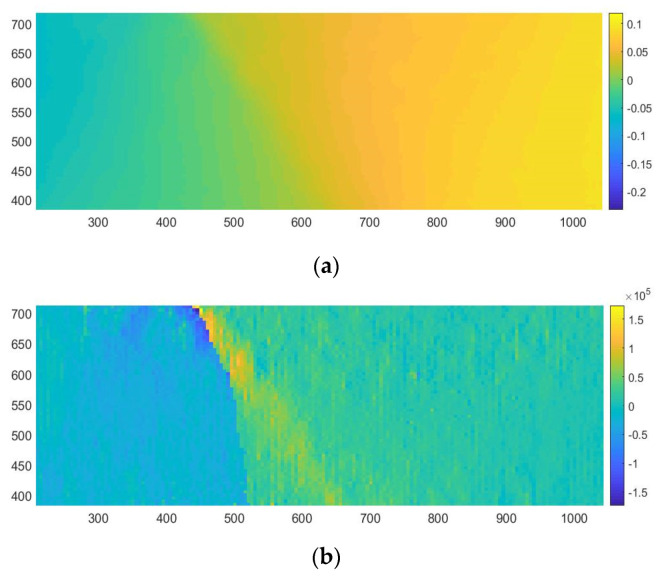
Real uniaxial tensile experiment of a small-scale specimen. (**a**) The measured horizontal displacement field and (**b**) the measured horizontal strain field.

Figure 18 shows the measured horizontal displacement and strain fields according to the images in Figure 17a,b. As shown in Figure 18a, the displacement gradually increased away from the yellow and blue border at an oblique 45-degree angle. We assumed that the displacement value from the yellow and blue border to the right is positive; otherwise it is negative. The displacement value was an accumulation process, and the displacement value at the far right of the red area was about 0.1 mm. It can be seen from Figure 18b that the strains near the border were larger than others. The result coincided exactly with Figure 17c,d, and the specimen finally broke at this border.

**Figure 19:**
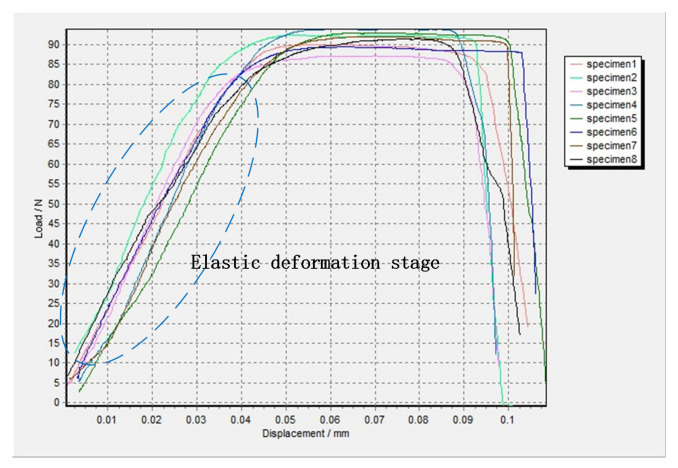
Displacement and load curve of specimens.

The uniaxial tensile experiments of T2 pure copper thin specimens with the same specimen size and experimental conditions were repeated eight times. While the computer received the load data, the two cameras also collected images of the specimen. We chose the narrow band region in the middle of the specimen (excluding the arc transition part) as the area to be calculated, which is similar to the red area in Figure 17a. The displacements of the marked points were calculated by the 3D-DIC method, and the interpolation of the two average displacements of the marked points on the right and left border of the selected narrow band region was recognized as the elongation of the narrow band region. The LVDT data was not used as the elongation of the specimen, because the data was the relative displacement between the two yokes, including the deformation of the yokes, the arc transition of the specimen, and clamping. This is why we used the 3D-DIC method to measure the true elongation of the specimen. The displacement load curves are shown in Figure 19. It can be seen that there are no yield stages in stretching processes of the small-scale T2 pure copper specimens. When the loads of the specimens were less than 8 0N, the specimens were in the elastic deformation stage. After the loads reached about 90 N, they were maintained for a period of time while the amounts of deformation increased. When the specimens were close to breaking, the loads dropped rapidly until the specimens break. As shown in Figure 19, the maximum load of the eight specimens was about 95 N, and the tensile strength of the specimen was about 475 MPa. The average value of the elastic modulus of the eight specimens was taken as the elastic modulus of the T2 pure copper at this size. Additionally, the calculated average elastic modulus was 188 GPa. The standard tensile strength and elastic modulus of T2 pure copper were 217 MPa and 110-128 GPa, respectively, with the specimen thickness of at least 0.2 mm [44]. It is easy to find that the mechanical properties of specimens of the same material with different physical sizes may be different. Therefore, we could not scale the mechanical properties according to the physical size.

## 5. Conclusions

In this paper, a testing system was proposed to measure the full-field strain and mechanical properties of small-scale specimens. A stereo light microscope SZX07 combined with two industrial cameras formed the imaging system employed to obtain the information of shape and full-field strain. Additionally, the micro tensile machine was used to clamp the small-scale specimen and produce very small deformations. Numerical and real experiments were performed to verify the testing system built and discussions were presented in the paper. All conclusions can be summarized as follows: (1) a testing system for investigating the mechanical properties of small-scale specimens based on three-dimensional (3D) microscopic digital image correlation (DIC) combined with a micro tensile machine is proposed; (2) the designed micro tensile machine can really stretch the specimen in-situ at a very small speed, which is convenient for observing the entire deformation process of the full-field; (3) in calibration computation, the reprojection error is smaller when the computer coordinates of the feature points are corrected; and (4) the error due to an undermatched shape function is far larger than that of an overmatched shape function. Moreover, the value of the correlation coefficient is reasonable as a criterion for selecting the shape function type.

## Figures and Tables

**Table 1:** Main performance parameters of the tensile machine.

Parameter	Reference Value
Tensile load	0–200 N
Straightness of load	0.21%
Displacement	−12 ± 12 mm
Straightness of LVDT	0.11%
Displacement speed	0.07 um/s to about 2 um/s
Specimen dimensions (length × width × thickness)	60 mm × 8 mm × 4 mm

**Table 2:** Parameters of the imaging system with the modified method.

Parameter	Camera 1			Camera 2		
f/*mm*	113.8105			96.1846		
ϑ	0.0056			0.0031		
$s_{x}$	1.0045			1.0049		
u_0_/pixel	558.4520			616.7733		
v_0_/pixel	545.0547			491.4351		
Rotation matrix	0.9954	0.0310	−0.0905	0.9914	0.0260	0.1286
	−0.0317	0.9995	0.0010	−0.0255	0.9997	−0.0033
	0.0904	0.0039	0.9959	−0.1287	−0.0004	0.9917
Translation vector	−11.2157	−11.3306	175.9052	−13.0778	−11.3371	148.9680

**Table 3:** Reprojection errors of Camera 1 using five calibration methods.

Method	Reprojection Error (pixels)
Zhang’s method	0.4218
Traditional two-step method	0.3909
WRAC without initial value	0.2860
WRAC with initial value	0.1285
Amplified WRAC method	0.0966
